# An update on the prevalence and management of Bertolotti's syndrome

**DOI:** 10.3389/fsurg.2024.1486811

**Published:** 2024-12-12

**Authors:** Taylor R. Rakauskas, Shannon Gallup, Ali A. Mohamed, Jude Nasice, Benjamin Westerhaus

**Affiliations:** ^1^ Charles E. Schmidt College of Medicine, Florida Atlantic University, Boca Raton, FL, United States; ^2^ Cantor Spine Center at the Paley Institute, West Palm Beach, FL, United States

**Keywords:** Bertolotti's syndrome, lumbosacral transitional vertebrae, low back pain, Castellvi classification, Onyiuke, Jenkins

## Abstract

Bertolotti's Syndrome, a subset of lumbosacral transitional vertebrae (LSTV), is one cause of chronic low back pain (LBP), and a commonly overlooked differential diagnosis. The incidence of Bertolotti's Syndrome has been underestimated in the past and is common in those of younger ages around 30–40. Although diagnostics, imaging methods, and treatment algorithms have been improved in the past few years, there is no gold standard and more long-term, prospective research is needed. The purpose of this mini-review is to increase awareness of Bertolotti's Syndrome, discuss recent advancements in treatment algorithms, and highlight current gaps in the literature.

## Introduction

Low back pain (LBP) is a common health problem that represents a global burden to patients, healthcare, and society. LBP affects over 13% of the population with the incidence increasing with age and remains a leading cause of disability and health care visits (1–3). Bertolotti's Syndrome is a commonly overlooked probable cause of chronic LBP associated with lumbosacral transitional vertebra (LSTV), a congenital anatomical anomaly of the lumbosacral spine where an enlarged transverse process of the lowest lumbar vertebra (L5) fuses with the first sacral vertebra (S1) to various degrees. It has been proposed that incomplete articulation between the transverse process and the sacral join can cause arthritic changes that contribute to pain (4). The spinal variation seen in Bertolotti's is accompanied by changes in the mechanics and forces through the lumbosacral spine akin to adjacent segment disease. Therefore, there is increased likelihood of herniations at the L4/5 level above the transitional vertebrae, and a decreased risk of herniation below the vertebrae (5).

The definitive diagnosis of Bertolotti's Pain Syndrome requires LSTV concomitant with LBP and is difficult to implicate due to low awareness and lack of inclusion in various diagnostic algorithms for the common complaint of LBP. Bertolotti's syndrome can be difficult to disgnose due to symptom overlap with many common causes of LBP, including herniated discs, spondylosis, stenosis, SI joint instability and facet arthritis. Symptom duration lasts for an average of 41.4 months prior to diagnosis, contributing to decreased quality of life and emotional wellbeing (6). There has been a steady increase in Bertolotti's syndrome among the literature, with a total of 118 articles and 419 patients as of September 2022 (6). Despite this upward trend and Bertolotti's Syndrome being described for the first time in 1917, a comprehensive treatment algorithm is still debated. Before this can be explored, we have completed this narrative to discuss recent treatment advancements, highlight research gaps, and increase awareness of Bertolotti's Syndrome.

### Search strategy

For this mini-review, a systematic search strategy per PRISMA guidelines was used across multiple academic databases, including PubMed/MEDLINE, Embase, and Web of Science. Articles were identified using the search terms “Bertolotti's syndrome,” “lumbosacral transitional vertebra,” and “LSTV”. We included studies reporting on treatment of Bertolotti's syndrome. We also included previous reviews to find additional sources.

### Epidemiology

The incidence of LSTV in the general population is widely debated and, as a result, the relationship between LSTV and LBP is not well defined. Although early studies deemed LSTV to be a rare anatomical variation, revised classification and improved imaging suggest the occurrence to be more common. Recent studies on the prevalence of LSTV among the general population and the underdiagnosis of Bertolotti's Syndrome among this population suggests that this condition may be less rare than once thought (7, 8). Some studies estimate prevalence of LSTV as high as 10%–20% in the general population and significantly greater in patients with symptomatic lumbar spine pain (Bertolotti's syndrome) (4, 9, 10). It has been shown to be more common in men than women (11). One study showed a prevalence of 26.8% from a collection size of 500. The high variability in the prevalence may be due patient selection bias or restricted age range, however studies consistently show no significant differences among the sexes (12).

The diagnosis of Bertolotti's syndrome is often delayed because the clinical picture can mimic many common conditions. Patients may complain of insidious, atraumatic lower back pain associated with stiffness. They may or may not have tenderness, either in a focal or non-focal location. This picture may mimic common conditions such as lumbar muscle strain, lumbar spondylosis, facet arthritis, lumbar radiculopathy, degenerative disc disease and neurogenic claudication. A thorough history and physical, including reflexes, provocative maneuvers, sensation, and muscle strength should be performed.

It is important to note the relationship between Bertolotti's syndrome and age. The average patient is between 30 and 50 years old but it can be seen in adolescents or older patients as well. Among a cohort of 268 patients, the mean age came out to 47.7 years, but this is likely to be an overestimate of the true mean due to missed diagnosis (6). While the prevalence of LSTV has not been associated with increasing age, the severity of Bertolotti's is significantly affected (13). The etiology of Bertolotti's syndrome highlights the importance of an early diagnosis for control of pain and the need to include LSTV in the differential for LBP.

### Anatomy and classification

The most commonly used classification of LSTV follows the Castellvi system and is composed of four types (14). Type I involves unilateral (Ia) or bilateral (Ib) enlarged and dysplastic transverse processes greater than 9 mm. Type II (unilateral: IIa, bilateral: IIb) exhibits additional pseudoarticulation of the enlarged transverse process resulting in partial sacralization or lumbarization, which is suggested to be the leading cause of Bertolotti's Syndrome. Complete sacralization/lumbarization classifies Type III (unilateral: IIIa, bilateral: IIIb) and a mixture of complete and incomplete sacralization is seen with Type IV. Variability exits among prevelence studies among the different types, likely due to small sample size. Type I and type II are the most common anomalies accounting for 40% of cases each. Type III accounts for 12% of cases and type IV is the rarest accounting for approximately 5% of cases (11).

Diagnosis of Bertolotti's syndrome relies heavily on imaging (x-ray, MRI, CT). The Castellvi classification is useful for radiographic diagnosis but does not reveal a specific source of the pain and may limit the ability to predict need for treatment (15, 16). The Castellvi classification excludes 2 types of anatomic variants: the prominent anatomic side and the potential transverse process and iliac crest contact. Furthermore, the credibility of this classification has been called into question following a recent study that reported a sensitivity of 76%–84%, but accuracy of only 53%–58% in identifying the structural anomaly on plain radiograph (17). A contrasting classification system proposed by Knopf et al. known as the Onyiuke scale takes into account the patients' symptoms, rather than just the visible anomalies found on imaging (15). The scale includes four grades, similar to Castellvi, however each grade is divided among a and b subtypes due to the involvement of a single level LSTV vs. multiple levels respectively. This classification system allows us to better understand how severe the disease is based on clinical symptoms, even if it is not visible on radiography.

Another newly proposed Jenkins classification of Bertolotti syndrome considers the distance between the transverse processes, whereas the original Castellvi classification only considers the height of the transverse processes (16). This difference in focus allows the Jenkins classification to better predict the symptoms that a patient is likely to experience as well as guide treatment options when conservative measures fail. Patients with Type 1 and 2 according to the Jenkins Criteria respond well to resection procedures whereas patients with Type 3 and 4 respond well to fusion procedures. This difference in consideration combines the advantages of the Castellvi and Onyiuke classification as well as the additional benefit of treatment guidance (Table 1).

**Table 1:** Comparison of the different classification systems for Bertolotti's syndrome.

Classification	Grading	Clinical Advantage	Disadvantage
Castellvi	Ia–IVb. (a) denotes unilateral, (b) denotes bilateral involvement	Most used classification. Useful for radiographic diagnosis. Considers the height of the transverse processes	Does not convey the source of pain and is limited in communicating treatment options. Excludes some anatomic variants. Relies on imaging. Accuracy low on plain radiograph
Onyiuke	Ia–IVb. (a) denotes single vertebral level involvement, (b) denotes multiple vertebral level involvement	Considers clinical symptoms along with radiographic evidence	Less commonly used. Limited ability to guide treatment options
Jenkins	I-IV	Considers radiographic evidence and patient symptomatology. Considers height and distance of transverse processes. May guide treatment options	Newly published (2023), less commonly used and less validated

### Pathophysiology

The presence of LSTV leads to changes in spinal biomechanics. Disruption of the normal skeletal anatomy is argued to cause the development of LBP, although the specific source has not yet been identified. Prior research suggests four possible causes: hypomobility at the L5/S1 level with hypermobility at superior levels, degenerative changes, foraminal stenosis, and contralateral facet joint arthrosis (18).

Arthritis changes at the pseudoarticulation and disc degeneration can also contribute to symptoms.

Facet joint degeneration has been found to occur at higher rates in affected patients above the LSTV, typically at the L5/S1 level (19). Additionally, degeneration of the LSTV and sacrum articulation, as well as lumbar spine degeneration, has been reported as a contributing cause of LBP in the presence of LSTV (9, 20). Other reported arthritic changes that are suggestively causative include degenerative osteophytic fusion, pseudoarticulation, and osseous fusion, depending on the LSTV type (14, 20). A key distinction between the joint of a patient with the arthritic changes seen in LSTV and a typical joint is the painful and inflammatory presentation caused by the loss of joint lining, cartilage, and synovial fluid.

Hypomobility of the transitional vertebrae with subsequent hypermobility at superior levels, significantly L2–L3, can result in protrusion at a younger age than those without LSTV (9, 21).

Subsequently, disc degeneration in patients with LSTV have similarly been shown to occur at more frequent rates as well as at younger age and may also be responsible for the identified discogenic pain. Disc degeneration rates follow a similar trend as observed with disc herniations, with higher rates occurring at the level immediately above the LSTV in affected patients (19, 21, 22). Additionally, rates of degeneration at the level above the LSTV occur at higher rates than between the sacrum and transitional vertebra.

Stenosis of the spinal canal and foramina constitutes the final suggestive cause of LBP in patients with LSTV. Likely caused by the associated facet joint degeneration seen in LSTV, intervertebral foramen stenosis has been identified in many cases of Bertolotti syndrome (19, 22). As seen in cases of facet joint arthrosis and disc herniation, stenosis has also been demonstrated to occur more frequently at the level above the LSTV (22).

### Conservative treatment

Treatment options for Bertolotti's syndrome range from conservative methods to surgical intervention. Like other causes of LBP, conservative treatments are tried before progressing to more invasive options. Many of the studies on conservative therapies are reported on a case study basis and more randomized, large scale investigations represent a gap in our current knowledge of management.

Initial treatment to consider are activity modification and pharmacotherapy including NSAIDs and muscle relaxants. Next to be considered are physical therapy and steroid injections. Physical therapy is aimed at lumbosacral manipulation and mobility. Son et al, 2016 found that transforaminal epidural injection is less effective in patients with Bertolotti's syndrome compared to patients with lumbar disc herniation alone (23). Another prospective clinical trial showed that transforaminal epidural steroid injections to treat LBP were less effective for patients with sacralization vs. without at 3 months follow-up (24). Combined injections of lidocaine and cortisone have been shown to most effectively reduce pain, however the majority of patients required eventual surgical intervention (25). Anesthetic and cortisone injections may be used diagnostically to determine the anatomical source of the pain, although some patients may not receive diagnostic local injections prior to surgical treatment. Case reports have demonstrated the effectiveness of radiofrequency ablation and the placement of bipolar radiofrequency strip thermal lesions (26). *need more here*

Chronic LBP affects many aspects of a person's life including work, interpersonal relationships, and psychological health. The psychiatric burden of chronic lower back pain should not be ignored and care should be taken to investigate and treat underlying depression. It is specifically important to rule out Bertolotti syndrome in younger patients under 35 with persistent LBP due to its congenital origin and possibly high prevalence.

### Surgical treatment

Beyond conservative therapy, surgical management is an option for unresolved cases. The route of surgical treatment is largely based on the LSTV classification and origin of pain. Surgical treatment is approached when conservative measures fail, however, given the progressive nature of the disease, it is important to consider surgery in symptomatic young patients (27).

The most reported surgical treatment methods include transverse processectomy (resection) and fusion of the L4-S1 or L5-S1 vertebral levels. Between fusion and resection of transverse segments, fusion is proposed to have several advantages although both have shown significant short-term pain improvement (28). First, it generally has less complications, lower blood loss, and shorter recovery times. Second, fusion is an established treatment that has been shown to reduce pain in similar degenerative articulation phenomena such as degenerative scoliosis. Resection of the pseudoarticulation may be suitable for patients with pain originating from the pseudo joint (29). However, resection potentially adds mobility to areas of the joint that have been previously stabilized, leading to potential novel movement patterns the body may not be adapted to by destabilizing a previously immobile joint.

Santavirta et al. (30) surgically treated 8 patients with resection of the pseudoarticulation and 8 patients with fusion. They found 10/16 patients had LBP improvement with no statistically significant difference between the groups. A more recent study by Mikula et al. (28) supports the conclusion of both resection and fusion reducing pain in the short-term. However, fusion showed to be superior beyond 12 months post-op, with a pain improvement of 78% compared to 28% for the resection group.

Long spinal fusion has become increasingly popular for the correction of LSTV, however we do see large rates of pseudoarthrosis and need for revision (31, 32). Extension of the fixation instrument to the pelvis has become common to avoid these complications, but spinopelvic fixation methods present issues of their own with certain screws leading to postoperative pain with protrusion. A recent study investigated the safety of various spinopelvic screws, concluding the greatest safety and least vascular damage with S1-pedicle screws (33). This finding agrees with other studies and emphasizes the continuous refinement of LSTV fusion techniques and the need for further investigation of fixation safety across different classifications of Bertolotti's Syndrome.

The Jenkins classification of LSTV described above has shown early success in guiding successful treatment for patients who fail conservative therapy. Jenkins et al. (15) described the outcomes of 56 patients who underwent the proposed treatments according to the Jenkin's criteria. Among patients with Type I anomaly who underwent the suggested resection, 85% had improvement of their symptoms and 54% experienced a reduction in their pain level to greater than 50% of baseline. In Type 2 patients, 88% of those who underwent fusion saw some improvement and 72%) had greater than 50% reduction in their baseline pain. In Type 4 patients, 86% has reduction of pain with unilateral fusion, with durable benefit at 2 years. In all patients who had hip pain preoperatively (*n* = 27), 21 (78%) had improvement of hip pain postoperatively (34). Although larger studies are needed to further validate the Jenkins criteria, this early data is optimistic about successfully guiding interventional treatment options.

Minimally invasive techniques for a number of spinal surgeries including Bertolotti's have been proposed in the recent literature (35, 36). A number of case studies have demonstrated the use of minimally invasive approach for resection and fusion for Bertolotti syndrome, demonstrating the benefits of minimally invasive procedures for patients with persistent pain despite multiple failed treatments (37). Li et al. performed a study on 7 patients involving endoscopic resection of the pseudoarticulation with a paramedian tubular approach that showed significantly reduced back pain in 5/7 subjects, but a long-term return of symptoms after initial relief in the remaining 2 patients (38). They improved their method from a previous case report throughout this study, most notably by reducing cauterization and replacing it with sharp dissection and bone wax.

Overall studies on the treatment of Bertolotti's syndrome often lack substantial methodological quality based on the methodological index for non-randomized studies (MINORS) criteria. Control groups are often absent and only 2 papers currently use the full 12 items (6).

## Conclusion

Bertolotti's syndrome is relatively rare, but an important condition to include in the differential of lower back pain. The clinical management focuses on symptom relief and improving patients' quality of life. There is clear importance in studying Bertolotti's syndrome to fully understand its role in chronic lower back pain. Enhanced understanding could inform future treatments and maximize patient outcomes. Further research is necessary to enhance our understanding of Bertolotti's syndrome, improve treatment plans, and maximize patient outcomes.
